# A systematic review of the content of critical appraisal tools

**DOI:** 10.1186/1471-2288-4-22

**Published:** 2004-09-16

**Authors:** Persis Katrak, Andrea E Bialocerkowski, Nicola Massy-Westropp, VS Saravana Kumar, Karen A Grimmer

**Affiliations:** 1 Centre for Allied Health Evidence: A Collaborating Centre of the Joanna Briggs Institute, City East Campus, University of South Australia, North Terrace, Adelaide, 5000, Australia; 2 School of Physiotherapy, The University of Melbourne, Melbourne, 3010, Australia

## Abstract

**Background:**

Consumers of research (researchers, administrators, educators and clinicians) frequently use standard critical appraisal tools to evaluate the quality of published research reports. However, there is no consensus regarding the most appropriate critical appraisal tool for allied health research. We summarized the content, intent, construction and psychometric properties of published, currently available critical appraisal tools to identify common elements and their relevance to allied health research.

**Methods:**

A systematic review was undertaken of 121 published critical appraisal tools sourced from 108 papers located on electronic databases and the Internet. The tools were classified according to the study design for which they were intended. Their items were then classified into one of 12 criteria based on their intent. Commonly occurring items were identified. The empirical basis for construction of the tool, the method by which overall quality of the study was established, the psychometric properties of the critical appraisal tools and whether guidelines were provided for their use were also recorded.

**Results:**

Eighty-seven percent of critical appraisal tools were specific to a research design, with most tools having been developed for experimental studies. There was considerable variability in items contained in the critical appraisal tools. Twelve percent of available tools were developed using specified empirical research. Forty-nine percent of the critical appraisal tools summarized the quality appraisal into a numeric summary score. Few critical appraisal tools had documented evidence of validity of their items, or reliability of use. Guidelines regarding administration of the tools were provided in 43% of cases.

**Conclusions:**

There was considerable variability in intent, components, construction and psychometric properties of published critical appraisal tools for research reports. There is no "gold standard' critical appraisal tool for any study design, nor is there any widely accepted generic tool that can be applied equally well across study types. No tool was specific to allied health research requirements. Thus interpretation of critical appraisal of research reports currently needs to be considered in light of the properties and intent of the critical appraisal tool chosen for the task.

## Background

Consumers of research (clinicians, researchers, educators, administrators) frequently use standard critical appraisal tools to evaluate the quality and utility of published research reports [1]. Critical appraisal tools provide analytical evaluations of the quality of the study, in particular the methods applied to minimise biases in a research project [2]. As these factors potentially influence study results, and the way that the study findings are interpreted, this information is vital for consumers of research to ascertain whether the results of the study can be believed, and transferred appropriately into other environments, such as policy, further research studies, education or clinical practice. Hence, choosing an appropriate critical appraisal tool is an important component of evidence-based practice.

Although the importance of critical appraisal tools has been acknowledged [1,3-5] there appears to be no consensus regarding the 'gold standard' tool for any medical evidence. In addition, it seems that consumers of research are faced with a large number of critical appraisal tools from which to choose. This is evidenced by the recent report by the Agency for Health Research Quality in which 93 critical appraisal tools for quantitative studies were identified [6]. Such choice may pose problems for research consumers, as dissimilar findings may well be the result when different critical appraisal tools are used to evaluate the same research report [6].

Critical appraisal tools can be broadly classified into those that are research design-specific and those that are generic. Design-specific tools contain items that address methodological issues that are unique to the research design [5,7]. This precludes comparison however of the quality of different study designs [8]. To attempt to overcome this limitation, generic critical appraisal tools have been developed, in an attempt to enhance the ability of research consumers to synthesise evidence from a range of quantitative and or qualitative study designs (for instance [9]). There is no evidence that generic critical appraisal tools and design-specific tools provide a comparative evaluation of research designs.

Moreover, there appears to be little consensus regarding the most appropriate items that should be contained within any critical appraisal tool. This paper is concerned primarily with critical appraisal tools that address the unique properties of allied health care and research [10]. This approach was taken because of the unique nature of allied health contacts with patients, and because evidence-based practice is an emerging area in allied health [10]. The availability of so many critical appraisal tools (for instance [6]) may well prove daunting for allied health practitioners who are learning to critically appraise research in their area of interest. For the purposes of this evaluation, allied health is defined as encompassing "...all occasions of service to non admitted patients where services are provided at units/clinics providing treatment/counseling to patients. These include units primarily concerned with physiotherapy, speech therapy, family panning, dietary advice, optometry occupational therapy..." [11].

The unique nature of allied health practice needs to be considered in allied health research. Allied health research thus differs from most medical research, with respect to:

• the paradigm underpinning comprehensive and clinically-reasoned descriptions of diagnosis (including validity and reliability). An example of this is in research into low back pain, where instead of diagnosis being made on location and chronicity of pain (as is common) [12], it would be made on the spinal structure and the nature of the dysfunction underpinning the symptoms, which is arrived at by a staged and replicable clinical reasoning process [10,13].

• the frequent use of multiple interventions within the one contact with the patient (an occasion of service), each of which requires appropriate description in terms of relationship to the diagnosis, nature, intensity, frequency, type of instruction provided to the patient, and the order in which the interventions were applied [13]

• the timeframe and frequency of contact with the patient (as many allied health disciplines treat patients in episodes of care that contain multiple occasions of service, and which can span many weeks, or even years in the case of chronic problems [14])

• measures of outcome, including appropriate methods and timeframes of measuring change in impairment, function, disability and handicap that address the needs of different stakeholders (patients, therapists, funders etc) [10,12,13].

## Methods

### Search strategy

In supplementary data [see additional file 1].

### Data organization and extraction

Two independent researchers (PK, NMW) participated in all aspects of this review, and they compared and discussed their findings with respect to inclusion of critical appraisal tools, their intent, components, data extraction and item classification, construction and psychometric properties. Disagreements were resolved by discussion with a third member of the team (KG).

Data extraction consisted of a four-staged process. First, identical replica critical appraisal tools were identified and removed prior to analysis. The remaining critical appraisal tools were then classified according to the study design for which they were intended to be used [1,2]. The scientific manner in which the tools had been constructed was classified as whether an empirical research approach has been used, and if so, which type of research had been undertaken. Finally, the items contained in each critical appraisal tool were extracted and classified into one of eleven groups, which were based on the criteria described by Clarke and Oxman [4] as:

• Study aims and justification

• Methodology used, which encompassed method of identification of relevant studies and adherence to study protocol;

• Sample selection, which ranged from inclusion and exclusion criteria, to homogeneity of groups;

• Method of randomization and allocation blinding;

• Attrition: response and drop out rates;

• Blinding of the clinician, assessor, patient and statistician as well as the method of blinding;

• Outcome measure characteristics;

• Intervention or exposure details;

• Method of data analyses;

• Potential sources of bias; and

• Issues of external validity, which ranged from application of evidence to other settings to the relationship between benefits, cost and harm.

An additional group, "miscellaneous", was used to describe items that could not be classified into any of the groups listed above.

### Data synthesis

Data was synthesized using MS Excel spread sheets as well as narrative format by describing the number of critical appraisal tools per study design and the type of items they contained. Descriptions were made of the method by which the overall quality of the study was determined, evidence regarding the psychometric properties of the tools (validity and reliability) and whether guidelines were provided for use of the critical appraisal tool.

## Results

One hundred and ninety-three research reports that potentially provided a description of a critical appraisal tool (or process) were identified from the search strategy. Fifty-six of these papers were unavailable for review due to outdated Internet links, or inability to source the relevant journal through Australian university and Government library databases. Of the 127 papers retrieved, 19 were excluded from this review, as they did not provide a description of the critical appraisal tool used, or were published in languages other than English. As a result, 108 papers were reviewed, which yielded 121 different critical appraisal tools [1-5,7,9,15-102,116].

### Empirical basis for tool construction

We identified 14 instruments (12% all tools) which were reported as having been constructed using a specified empirical approach [20,29,30,32,35,40,49,51,70-72,79,103,116]. The empirical research reflected descriptive and/or qualitative approaches, these being critical review of existing tools [40,72], Delphi techniques to identify then refine data items [32,51,71], questionnaires and other forms of written surveys to identify and refine data items [70,79,103], facilitated structured consensus meetings [20,29,30,35,40,49,70,72,79,116], and pilot validation testing [20,40,72,103,116]. In all the studies which reported developing critical appraisal tools using a consensus approach, a range of stakeholder input was sought, reflecting researchers and clinicians in a range of health disciplines, students, educators and consumers. There were a further 31 papers which cited other studies as the source of the tool used in the review, but which provided no information on why individual items had been chosen, or whether (or how) they had been modified. Moreover, for 21 of these tools, the cited sources of the critical appraisal tool did not report the empirical basis on which the tool had been constructed.

### Critical appraisal tools per study design

**Figure 1:**
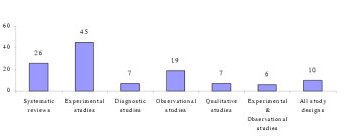
Number of critical appraisal tools per study design [1,2]

Seventy-eight percent (N = 94) of the critical appraisal tools were developed for use on primary research [1-5,7,9,18,19,25-27,34,37-41], while the remainder (N = 26) were for secondary research (systematic reviews and meta-analyses) [2-5,15-36,116]. Eighty-seven percent (N = 104) of all critical appraisal tools were design-specific [2-5,7,9,15-90], with over one third (N = 45) developed for experimental studies (randomized controlled trials, clinical trials) [2-4,25-27,34,37-73]. Sixteen critical appraisal tools were generic. Of these, six were developed for use on both experimental and observational studies [9,91-95], whereas 11 were purported to be useful for any qualitative and quantitative research design [1,18,41,96-102,116] (see Figure 1, Table 1).

**Table 1:** Summary of tools sourced in this review.

**Research design focus of critical appraisal tools**		**Critical appraisal tools with summary scores**	
**Secondary studies**	Systematic reviews/meta-analyses [2-5,15-36,116]	**All study designs [1,18,41,96-102,116]**	Summary score [18,41,96,97,116]
**Primary studies**	Experimental studies [2-4,19,25-27,34,37-73]		No summary score [1,98-102]
	Diagnostic studies [19,74-79]	**Experimental studies**	Summary score [19,37-59]
	Observational studies [2,3,7,19,25,66,72,80-86]		No summary score [2-4,25,27,28,34,60-73]
	Qualitative studies [9,26,66,87-90]	**Diagnostic studies**	Summary score [16,74-77]
	Experimental & Observational studies [9,91-102]		No summary score [78,79]
		**Qualitative studies**	Summary score [87]
			No summary score [9,26,66,88-90]
		**Experimental and observational studies**	Summary score [91-93]
			No summary score [9,94,95]

### Critical appraisal items

One thousand, four hundred and seventy five items were extracted from these critical appraisal tools. After grouping like items together, 173 different item types were identified, with the most frequently reported items being focused towards assessing the external validity of the study (N = 35) and method of data analyses (N = 28) (Table 2). The most frequently reported items across all critical appraisal tools were:

**Table 2:** The type and number of component items contained in critical appraisal tools per study design.

**Type of items**	**Design-specific critical appraisal tool components**					**Generic critical appraisal tool components**		**Total**
		
	**Systematic reviews**	**Experimental studies**	**Diagnostic studies**	**Observational studies**	**Qualitative studies**	**Exp & Obs^a ^studies**	**All study designs**	
Study aims and justification	35	27	5	18	17	4	11	117
Methodology used	38	1	0	0	0	0	1	40
Sample selection	30	62	12	37	10	10	14	175
Randomization	2	65	1	5	0	6	5	84
Attrition	4	59	3	23	0	8	8	105
Blinding	1	77	5	8	0	5	7	103
Outcome measure characteristics	41	46	3	33	2	9	19	153
Intervention	7	42	3	13	0	5	12	82
Data analyses	83	91	14	54	12	14	27	295
Bias	24	14	2	5	0	3	6	54
External validity	72	50	12	30	27	9	27	227
Miscellaneous	11	12	7	5	7	2	6	50
**Total**	348	546	67	331	75	75	143	1485

• Eligibility criteria (inclusion/exclusion criteria) (N = 63)

• Appropriate statistical analyses (N = 47)

• Random allocation of subjects (N = 43)

• Consideration of outcome measures used (N = 43)

• Sample size justification/power calculations (N = 39)

• Study design reported (N = 36)

• Assessor blinding (N = 36)

### Design-specific critical appraisal tools

#### Systematic reviews

Eighty-seven different items were extracted from the 26 critical appraisal tools, which were designed to evaluate the quality of systematic reviews. These critical appraisal tools frequently contained items regarding data analyses and issues of external validity (Tables 2 and 3).

**Table 3:** The type and number of guidelines accompanying critical appraisal tools per study design

**Type of critical appraisal tool**	**Type of guideline**					**Total number of critical appraisal tools**
		
	**Handbook/published paper**		**Accompanying explanation**		**Total**	
			
	**Number of tools**	**References**	**Number of tools**	**References**		
Systematic reviews	9	[2,4,15,20,25,28,29,331,36,116]	3	[16,26,27]	12	26
Experimental studies	10	[2,4,25,37,41,50,64-66,69]	6	[26,40,49,51,57,59]	16	45
Diagnostic studies	3	[74,75,76]	1	[79]	4	7
Observational studies	9	[2,25,66,80,84-87]	1	[83]	10	19
Qualitative studies	4	[9,87,89,90]	1	[26]	5	7
Experimental & Observational studies	2	[9,95]	1	[91]	3	6
All study designs	1	[100]	1	[102]	2	10
**Total**	**38**		**14**		**52**	**120**

Items assessing data analyses were focused to the methods used to summarize the results, assessment of sensitivity of results and whether heterogeneity was considered, whereas the nature of reporting of the main results, interpretation of them and their generalizability were frequently used to assess the external validity of the study findings. Moreover, systematic review critical appraisal tools tended to contain items such as identification of relevant studies, search strategy used, number of studies included and protocol adherence, that would not be relevant for other study designs. Blinding and randomisation procedures were rarely included in these critical appraisal tools.

#### Experimental studies

One hundred and twenty thirteen different items were extracted from the 45 experimental critical appraisal tools. These items most frequently assessed aspects of data analyses and blinding (Tables 1 and 2). Data analyses items were focused on whether appropriate statistical analysis was performed, whether a sample size justification or power calculation was provided and whether side effects of the intervention were recorded and analysed. Blinding was focused on whether the participant, clinician and assessor were blinded to the intervention.

#### Diagnostic studies

Forty-seven different items were extracted from the seven diagnostic critical appraisal tools. These items frequently addressed issues involving data analyses, external validity of results and sample selection that were specific to diagnostic studies (whether the diagnostic criteria were defined, definition of the "gold" standard, the calculation of sensitivity and specificity) (Tables 1 and 2).

#### Observational studies

Seventy-four different items were extracted from the 19 critical appraisal tools for observational studies. These items primarily focused on aspects of data analyses (see Tables 1 and 2, such as whether confounders were considered in the analysis, whether a sample size justification or power calculation was provided and whether appropriate statistical analyses were preformed.

#### Qualitative studies

Thirty-six different items were extracted from the seven qualitative study critical appraisal tools. The majority of these items assessed issues regarding external validity, methods of data analyses and the aims and justification of the study (Tables 1 and 2). Specifically, items were focused to whether the study question was clearly stated, whether data analyses were clearly described and appropriate, and application of the study findings to the clinical setting. Qualitative critical appraisal tools did not contain items regarding sample selection, randomization, blinding, intervention or bias, perhaps because these issues are not relevant to the qualitative paradigm.

#### Generic critical appraisal tools

##### Experimental and observational studies

Forty-two different items were extracted from the six critical appraisal tools that could be used to evaluate experimental and observational studies. These tools most frequently contained items that addressed aspects of sample selection (such as inclusion/exclusion criteria of participants, homogeneity of participants at baseline) and data analyses (such as whether appropriate statistical analyses were performed, whether a justification of the sample size or power calculation were provided).

##### All study designs

Seventy-eight different items were contained in the ten critical appraisal tools that could be used for all study designs (quantitative and qualitative). The majority of these items focused on whether appropriate data analyses were undertaken (such as whether confounders were considered in the analysis, whether a sample size justification or power calculation was provided and whether appropriate statistical analyses were preformed) and external validity issues (generalization of results to the population, value of the research findings) (see Tables 1 and 2).

### Allied health critical appraisal tools

We found no critical appraisal instrument specific to allied health research, despite finding at least seven critical appraisal instruments associated with allied health topics (mostly physiotherapy management of orthopedic conditions) [37,39,52,58,59,65]. One critical appraisal development group proposed two instruments [9], specific to quantitative and qualitative research respectively. The core elements of allied health research quality (specific diagnosis criteria, intervention descriptions, nature of patient contact and appropriate outcome measures) were not addressed in any one tool sourced for this evaluation. We identified 152 different ways of considering quality reporting of outcome measures in the 121 critical appraisal tools, and 81 ways of considering description of interventions. Very few tools which were not specifically targeted to diagnostic studies (less than 10% of the remaining tools) addressed diagnostic criteria. The critical appraisal instrument that seemed most related to allied health research quality [39] sought comprehensive evaluation of elements of intervention and outcome, however this instrument was relevant only to physiotherapeutic orthopedic experimental research.

### Overall study quality

**Figure 2:**
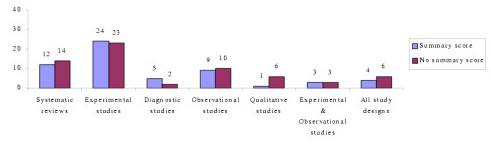
Number of critical appraisal tools with, and without, summary quality scores

Forty-nine percent (N = 58) of critical appraisal tools summarised the results of the quality appraisal into a single numeric summary score [5,7,15-25,37-59,74-77,80-83,87,91-93,96,97] (Figure 2). This was achieved by one of two methods:

• An equal weighting system, where one point was allocated to each item fulfilled; or

• A weighted system, where fulfilled items were allocated various points depending on their perceived importance.

However, there was no justification provided for any of the scoring systems used. In the remaining critical appraisal tools (N = 62), a single numerical summary score was not provided [1-4,9,25-36,60-73,78,79,84-90,94,95,98-102]. This left the research consumer to summarize the results of the appraisal in a narrative manner, without the assistance of a standard approach.

### Psychometric properties of critical appraisal tools

Few critical appraisal tools had documented evidence of their validity and reliability. Face validity was established in nine critical appraisal tools, seven of which were developed for use on experimental studies [38,40,45,49,51,63,70] and two for systematic reviews [32,103]. Intra-rater reliability was established for only one critical appraisal tool as part of its empirical development process [40], whereas inter-rater reliability was reported for two systematic review tools [20,36] (for one of these as part of the developmental process [20]) and seven experimental critical appraisal tools [38,40,45,51,55,56,63] (for two of these as part of the developmental process [40,51]).

### Critical appraisal tool guidelines

Forty-three percent (N = 52) of critical appraisal tools had guidelines that informed the user of the interpretation of each item contained within them (Table 2). These guidelines were most frequently in the form of a handbook or published paper (N = 31) [2,4,9,15,20,25,28,29,31,36,37,41,50,64-67,69,80,84-87,89,90,95,100,116], whereas in 14 critical appraisal tools explanations accompanied each item [16,26,27,40,49,51,57,59,79,83,91,102].

## Discussion

Our search strategy identified a large number of published critical appraisal tools that are currently available to critically appraise research reports. There was a distinct lack of information on tool development processes in most cases. Many of the tools were reported to be modifications of other published tools, or reflected specialty concerns in specific clinical or research areas, without attempts to justify inclusion criteria. Less than 10 of these tools were relevant to evaluation of the quality of allied health research, and none of these were based on an empirical research approach. We are concerned that although our search was systematic and extensive [104,105], our broad key words and our lack of ready access to 29% of potentially useful papers (N = 56) potentially constrained us from identifying all published critical appraisal tools. However, consumers of research seeking critical appraisal instruments are not likely to seek instruments from outdated Internet links and unobtainable journals, thus we believe that we identified the most readily available instruments. Thus, despite the limitations on sourcing all possible tools, we believe that this paper presents a useful synthesis of the readily available critical appraisal tools.

The majority of the critical appraisal tools were developed for a specific research design (87%), with most designed for use on experimental studies (38% of all critical appraisal tools sourced). This finding is not surprising as, according to the medical model, experimental studies sit at or near the top of the hierarchy of evidence [2,8]. In recent years, allied health researchers have strived to apply the medical model of research to their own discipline by conducting experimental research, often by using the randomized controlled trial design [106]. This trend may be the reason for the development of experimental critical appraisal tools reported in allied health-specific research topics [37,39,52,58,59,65].

We also found a considerable number of critical appraisal tools for systematic reviews (N = 26), which reflects the trend to synthesize research evidence to make it relevant for clinicians [105,107]. Systematic review critical appraisal tools contained unique items (such as identification of relevant studies, search strategy used, number of studies included, protocol adherence) compared with tools used for primary studies, a reflection of the secondary nature of data synthesis and analysis.

In contrast, we identified very few qualitative study critical appraisal tools, despite the presence of many journal-specific guidelines that outline important methodological aspects required in a manuscript submitted for publication [108-110]. This finding may reflect the more traditional, quantitative focus of allied health research [111]. Alternatively, qualitative researchers may view the robustness of their research findings in different terms compared with quantitative researchers [112,113]. Hence the use of critical appraisal tools may be less appropriate for the qualitative paradigm. This requires further consideration.

Of the small number of generic critical appraisal tools, we found few that could be usefully applied (to any health research, and specifically to the allied health literature), because of the generalist nature of their items, variable interpretation (and applicability) of items across research designs, and/or lack of summary scores. Whilst these types of tools potentially facilitate the synthesis of evidence across allied health research designs for clinicians, their lack of specificity in asking the 'hard' questions about research quality related to research design also potentially precludes their adoption for allied health evidence-based practice. At present, the gold standard study design when synthesizing evidence is the randomized controlled trial [4], which underpins our finding that experimental critical appraisal tools predominated in the allied health literature [37,39,52,58,59,65]. However, as more systematic literature reviews are undertaken on allied health topics, it may become more accepted that evidence in the form of other research design types requires acknowledgement, evaluation and synthesis. This may result in the development of more appropriate and clinically useful allied health critical appraisal tools.

A major finding of our study was the volume and variation in available critical appraisal tools. We found no gold standard critical appraisal tool for any type of study design. Therefore, consumers of research are faced with frustrating decisions when attempting to select the most appropriate tool for their needs. Variable quality evaluations may be produced when different critical appraisal tools are used on the same literature [6]. Thus, interpretation of critical analysis must be carefully considered in light of the critical appraisal tool used.

The variability in the content of critical appraisal tools could be accounted for by the lack of any empirical basis of tool construction, established validity of item construction, and the lack of a gold standard against which to compare new critical tools. As such, consumers of research cannot be certain that the content of published critical appraisal tools reflect the most important aspects of the quality of studies that they assess [114]. Moreover, there was little evidence of intra- or inter-rater reliability of the critical appraisal tools. Coupled with the lack of protocols for use, this may mean that critical appraisers could interpret instrument items in different ways over repeated occasions of use. This may produce variable results [123].

## Conclusions

Based on the findings of this evaluation, we recommend that consumers of research should carefully select critical appraisal tools for their needs. The selected tools should have published evidence of the empirical basis for their construction, validity of items and reliability of interpretation, as well as guidelines for use, so that the tools can be applied and interpreted in a standardized manner. Our findings highlight the need for consensus to be reached regarding the important and core items for critical appraisal tools that will produce a more standardized environment for critical appraisal of research evidence. As a consequence, allied health research will specifically benefit from having critical appraisal tools that reflect best practice research approaches which embed specific research requirements of allied health disciplines.

## Competing interests

No competing interests.

## Authors' contributions

PK Sourced critical appraisal tools

Categorized the content and psychometric properties of critical appraisal tools

AEB Synthesis of findings

Drafted manuscript

NMW Sourced critical appraisal tools

Categorized the content and psychometric properties of critical appraisal tools

VSK Sourced critical appraisal tools

Categorized the content and psychometric properties of critical appraisal tools

KAG Study conception and design

Assisted with critiquing critical appraisal tools and categorization of the content and psychometric properties of critical appraisal tools

Drafted and reviewed manuscript

Addressed reviewer's comments and re-submitted the article

## Pre-publication history

The pre-publication history for this paper can be accessed here:



## Supplementary Material

Additional File 1 Search Strategy. Click here for file
