# Supplementary material for: A systematic review of the content of critical appraisal tools
Source: BMC Med Res Methodol. 2004 Sep 16;4:22. doi: 10.1186/1471-2288-4-22 (PMC521688; doi:10.1186/1471-2288-4-22)
Supplement: Additional File 1 — Search Strategy. [file 1471-2288-4-22-S1.doc]

**Search Strategy**

***Statement of intent:*** All published critical appraisal tools were identified and reviewed for content and for relevance to allied health research. The references in the critical appraisal tool review (<http://www.ahrq.gov/clinic/epcsums/strengthsum.htm>) were used as a starting point, and critical appraisal instruments which have been published since then were also included. Design specific and generic instruments were all eligible for consideration.

# *Search Sources:* Electronic database and internet site searching was performed with no language or time limits. In addition to the reference above, other sources of critical appraisal tools were:

1. Trip database [http://www.tripdatabase.com](http://www.tripdatabase.com/)
2. Clinical Evidence [http://www.clinicalevidence.com](http://www.clinicalevidence.com/)
3. Physiotherapy Evidence Database [http://www.pedro.fhs.usyd.edu.au](http://www.pedro.fhs.usyd.edu.au/)
4. OT Seeker <http://www.otseeker.com/>
5. McMaster University Evidence-Based Practice Group http://www-fhs.mcmaster.ca/rehab/ebp
6. Medline
7. Embase
8. CINAHL
9. Current Contents
10. The Cochrane Library
11. Cochrane Database of Systematic Reviews (CDSR)
12. Database of Abstracts of Reviews of Effectiveness (DARE)
13. The Cochrane Controlled Trials Register (CCTR)
14. SIGN / NICE
15. NH&MRC
16. other clinical guideline and evidence-based practice sites identified from Google, Yahoo and MSN search engines
17. Reference lists and content experts were contacted to provide additional critical appraisal tools.

# Search Strategy

The search terms were broad, and consisted of words such as critical appraisal tools, critical appraisal, critical review form, systematic review form, appraisal of research methodology and research design review.

**Inclusion**

There was no date limit on the search. Critical appraisal tools were included if they:

1. were applicable to at least one research design (quantitative and qualitative research)
2. had clear and unambiguous criteria, and
3. could produce a numeric quality score.

# Exclusion

Critical appraisal instruments that were not published in full, or were not in English, were excluded. Tools that were used for appraisal of diagnostic instruments and clinical guidelines were excluded.
